# Peripheral Auditory Function in Tanzanian Children Living With HIV With Clinically Normal Hearing

**DOI:** 10.1001/jamanetworkopen.2023.3061

**Published:** 2023-03-15

**Authors:** Christopher E. Niemczak, Christin Ealer, Abigail Fellows, Albert Magohe, Jiang Gui, Catherine Rieke, Trent Nicol, Enica R. Massawe, Nina Kraus, Jay C. Buckey

**Affiliations:** 1 Department of Medicine, Dartmouth-Hitchcock Medical Center, Lebanon, New Hampshire; 2 Space Medicine Innovations Laboratory, Geisel School of Medicine at Dartmouth, Hanover, New Hampshire; 3 Muhimbili University of Health and Allied Sciences, Dar es Salaam, Tanzania; 4 Auditory Neuroscience Laboratory, Department of Communication Sciences, Northwestern University, Evanston, Illinois; 5 Auditory Neuroscience Laboratory, Departments of Communication Sciences, Neurobiology and Otolaryngology, Northwestern University, Evanston, Illinois

## Abstract

**Importance:**

Despite normal audiometry, adults living with HIV have lower distortion product otoacoustic emissions (DPOAEs) compared with HIV-negative controls, but the degree of these differences in children living with HIV is unknown. If subclinical auditory deficits are present, results could affect developmental outcomes in children living with HIV (CLWH).

**Objective:**

To compare DPOAEs and auditory brainstem responses (ABR) between 2 age- and sex-matched groups of younger children with normal audiometry, 1 infected with HIV and the other uninfected.

**Design, Setting, and Participants:**

Cohort study in an infectious disease center in Dar es Salaam, Tanzania. Participants included 340 Tanzanian children aged 3 to 9 years with clinically normal hearing, type A tympanograms bilaterally, and air-conduction thresholds of 20 dB HL or less from 0.5 to 8 kHz. Participants in the cohort repeated testing approximately every 6 months (approximately 2.2 sessions per participant) for a total of 744 total observations. Data were analyzed from March 2020 to January 2022.

**Main Outcomes and Measures:**

DPOAE amplitudes from 1.5 to 8 kHz using an f2 to f1 ratio of 1.2 and L1/L2 values of 65/55 dB sound pressure level and click-evoked ABR using a slow (21.1/s) and fast (61.1/s) click rate.

**Results:**

A total of 141 CLWH (70 female participants [49.3%]; mean [SD] age, 7.24 [1.67] years) and 199 HIV-negative individuals (99 female participants [49.7%]; mean [SD] age, 7.26 [1.44] years) participated in the study. The groups did not differ significantly in age, static immittance, or air-conduction thresholds. HIV status was independently associated with approximately 1.4 dB (95% CI, −3.28 to 0.30 dB) to 3.8 dB (95% CI, 6.03 to −1.99 dB) lower DPOAE amplitudes at 6 and 8 kHz bilaterally and 0.28 μV (95% CI, 0.01 to 0.33 μV) lower ABR wave V amplitudes in the right ear.

**Conclusions and Relevance:**

Consistent with previous findings in young adults, CLWH had slightly, but reliably, lower DPOAEs and ABR wave V amplitudes than HIV-negative controls. The magnitude of these differences was small, but results suggest an early and consistent association between HIV infection or treatment and outer hair cell and auditory brainstem responses in children as young as 3 years. These subclinical changes suggest tracking both auditory function and development outcomes in CLWH is warranted.

## Key Points

**Question:**

Are subclinical deficits present on distortion product otoacoustic emissions and auditory brainstem responses in children living with HIV with normal hearing sensitivity?

**Findings:**

In this cohort study of 340 participants, children living with HIV had slightly, but reliably, lower DPOAE amplitudes at 6 and 8 kHz and ABR wave V amplitudes compared with HIV-negative participants.

**Meaning:**

These results suggest a subtle, but early and consistent association between HIV infection or treatment and outer hair cell function and auditory brainstem responses in children perinatally infected with HIV.

## Teaser

This cohort study evaluates peripheral auditory function in Tanzanian children living with HIV with clinically normal hearing.

## Introduction

Both middle-aged and young adults infected with human immunodeficiency virus (HIV) show subclinical auditory deficits even with normal audiograms.^1,2,3,4,5,6,7,8^ Several clinical studies have shown peripheral auditory system abnormalities in people living with HIV (PLWH), including abnormal tympanograms, threshold audiograms, distortion product otoacoustic emissions (DPOAEs) tests, and auditory brainstem response results (ABRs),^2,4,5,9,10^ but no consistent pattern of peripheral hearing dysfunction in PLWH has emerged. Although our previous work focused on central auditory performance, we also compared peripheral auditory function in 2 age- and sex-matched groups of young adults (20-30 years) with clinically normal hearing with and without HIV.^2,8,11^ Results revealed that young adults living with HIV have slightly, but reliably, smaller distortion product otoacoustic emissions (DPOAEs) and auditory brainstem response (ABR) wave I amplitudes compared with matched HIV-negative controls. The degree of these differences was small and subclinical, but the results supported continued monitoring of peripheral auditory function in PLWH as they age. Analyzing the origin (ie, antiretroviral drug therapy, increased risk of infection due to HIV, and HIV itself) of these subclinical auditory deficits is ongoing, but assessing these findings in young children living with HIV (CLWH) may indicate whether these findings develop reliably and consistently in all age groups. Also, findings in young children are unlikely to be confounded by occupational factors such as noise exposure.

The current study expands previous work with young adults by comparing peripheral auditory function between young CLWH and age- and sex matched HIV-negative controls who all have clinically normal hearing.^12^ The data come from a longitudinal cohort of children in Tanzania who have been studied over the course of 3 years to assess if central auditory function is associated with literacy outcomes. This cohort has been tested with a comprehensive battery of both peripheral and central auditory tests and provides a unique opportunity to assess auditory function in CLWH in a low-to-middle-income country. If HIV infection or treatment does affect peripheral hearing, we might expect this cohort to exhibit subtle decreases on tests of peripheral auditory function as seen previously in young adults. We therefore hypothesized that normal hearing CLWH will show small but significant subclinical decreases in DPOAE and ABR amplitudes compared with age- and sex-matched HIV-negative children. Finding differences in young children living with HIV would suggest that these audiological results are a consistent finding due to HIV infection or treatment and are unlikely to be due to occupational factors. It would also prompt further analysis of educational and development outcomes in children with HIV due to differences in auditory function.

## Methods

### Recruitment

We recruited from a unique cohort of CLWH and HIV-negative children in Dar es Salaam, Tanzania, who have been performing central auditory, peripheral auditory, and cognitive testing approximately every 6 months for 3 years with unexpected interruptions due to COVID-19. This cohort study follows the Strengthening the Reporting of Observational Studies in Epidemiology (STROBE) reporting guideline. The research protocol was approved by the Committee for Protection of Human Subjects of Dartmouth College and the Research Ethics Committee of Muhimbili University of Health and Allied Sciences. A parent or guardian provided written informed consent for all children in the study.

### Study Procedures

Participants or an accompanying parent/guardian completed a series of questionnaires at the Infectious Disease Center in Dar es Salaam. The questionnaires gathered data on hearing ability and general health. The questionnaires covered ear pathology and asked about birth history, education, antiretroviral HIV treatment, gentamicin exposure, and the use of other ototoxic drugs.

Enrollment efforts for the overall project aimed at age- and sex-matching 2 groups of CLWH and HIV-negative individuals. All CLWH were perinatally infected with HIV. We further refined this matching procedure by selecting children between 3 and 9 years and using several data selection criteria. Children were excluded if they (1) had hearing sensitivity less than 25 dB HL from 0.5 to 8 kHz or abnormal middle ear function as indicated by Type B or C tympanograms; or (2) reported a positive history of ear drainage, concussion, neurological disease, ototoxic antibiotics (eg, gentamycin), noise exposure, or chemotherapy. This selection resulted in children with a mean (SD) of 2.18 (0.98) observations (ie, visits) per child (Table 1). Peripheral auditory assessment protocols have been previously described in Niemczak et al^12^ and summarized below.

**Table 1.** Characteristics and Statistical Comparisons Between CLWH and HIV-Negative Groups^a^

Demographic variable	Mean (SD)			*P* value for HIV-negative vs CLWH
	Overall cohort (N = 340)	HIV negative (n = 199)	CLWH (n = 141)	
Observations per participant	2.181 (0.98)	2.135 (1.13)	2.23 (0.75)	.03
Female sex, No. (%)	169 (49.6)	99 (49.7)	70 (49.3)	.69
Age, y	7.25 (1.51)	7.26 (1.44)	7.24 (1.67)	.97
Education, y	3.18 (1.60)	3.30 (1.62)	3.02 (1.53)	.10
Pure-tone average (0.5-4 kHz), dB HL				
Right ear	13.55 (4.85)	13.48 (4.65)	13.64 (5.14)	.75
Left ear	12.96 (5.15)	12.44 (5.16)	13.31 (5.12)	.04
Socioeconomic status^b^	0.094 (1.00)	0.14 (0.99)	−0.02 (1.04)	.60
Duration of HIV infection, y	NA	NA	5.58 (2.20)	NA
Currently on antiretroviral therapy, %	NA	NA	100	NA

Abbreviations: CLWH, children living with HIV; NA, not applicable.

^a^ A χ^2^ test was conducted on sex data and *t* tests comparing independent means were conducted on all continuous data. A significant difference between HIV groups was found for nonverbal IQ at a corrected significance level of *P* < .005.

### Otoscopy and Tympanometry

Otoscopy was performed on all participants to ensure a clear ear canal. Cerumen removal was performed if there was a substantial obstruction that could impede auditory measures. A Madsen Otoflex 100 (GN Otometrics) was used to perform tympanometry at 226 Hz. Measurements of ear canal volume, static admittance, tympanometric peak pressure, tympanometric width, and tympanogram type were collected. Type A tympanograms (including A_s_ and A_d_) were required for inclusion in this study (pressure limits from −100 to +50 daPa, and static admittance limits from 0.3 to 2.2 mmho).

### Audiometry

Pure-tone audiometry was completed using Creare LLC’s wireless automated hearing test system (WAHTS) controlled through a laptop. Every attempt was made to keep the testing room quiet, but the WAHTS system allowed for testing in rooms with suboptimal noise levels, as the device speakers are mounted in highly noise-attenuating ear cups. The attenuation provided by this headset is on par with a portable single-walled sound booth according to the relevant ANSI standards.^13^ Pure-tone air conduction thresholds were measured in octaves from 0.5 to 8 kHz using a modified Hughson-Westlake procedure.^14^ Conditioned play audiometry was implemented for participants that could not reliably complete the modified Hughson-Westlake procedure. A pure-tone average was calculated from 0.5 to 4.0 kHz.

### Distortion Product Otoacoustic Emissions

DPOAEs were collected with Creare LLC’s system at f2 values of 1.5, 2, 3, 4, 6, and 8 kHz using an f2 to f1 ratio of 1.2 and L1/L2 values of 65/55 dB sound pressure level as in our previous work.^2,12^ The f2 to f1 frequency pair was delivered for a minimum of 4s. After 4s, if the DPOAE level and averaged noise floor level difference was less than 10 dB signal-to-noise ratio, the frequency pair continued until either a DPOAE signal-to-noise ratio of 10 was reached or 10s had elapsed. The level of harmonic distortion for each system was determined using a Brüel and Kjær Type 4157 Ear Simulator/Artificial Ear (Brüel and Kjær). Because consistent DPOAE probe placement was important for achieving consistent results over time, a position check (frequency sweep) was presented in the ear canal before DPOAE testing.

### Auditory Brainstem Responses

An Intelligent Hearing Systems SmartEP (Miami, FL) was used to record ABR measurements in the right ear. The electrode montage consisted of the right earlobe as the inverting, ground at F_pz_, and the high forehead at F_z_ serving as the noninverting electrode. The stimuli were 100μs rarefaction clicks presented at a rate of 21.1/s (slow) or 61.1/s (fast) at 80 dB sound pressure level to the right ear. Two repetitions of each click were recorded and averaged (total 2000 sweeps). Responses were filtered from 0.1 to 1.5 kHz. The absolute latencies and amplitudes of waves, I, III, and V were measured from the 0 line.

### Statistical Analysis

Demographic characteristics of the groups were compared with *t* tests on continuous data and χ^2^ distribution tests for categorical data (eg, sex). Data were analyzed using linear mixed-effects models in MATLAB 2020a (Mathworks). The primary response variables were DPOAE amplitudes and ABR wave latencies and amplitudes. The model fixed effect was HIV status*,* and the random effect was participant variability of repeated measures. Including “participant” as a random factor over repeated observations allowed us to estimate fixed effects that replicated over time. Due to multiple comparisons, a corrected 2-sided *P*-value of .005 was implemented for all models. This correction was more liberal than a Bonferroni correction, but still controlled for excessive false-positive results. The primary hypothesis testing focused on the difference between HIV groups (HIV status). Data were analyzed from March 2020 to January 2022.

## Results

### Demographics

A total of 340 children were enrolled in the study. There were 141 enrolled in the CLWH group (mean [SD] age, 7.24 [1.67] years; 70 female participants [49.3%]) with normal hearing and tested a mean (SD) of 2.23 (0.75) times each. A total of 199 HIV-negative children (mean [SD] age, 7.26 [1.44] years; 99 female participants [49.7%]) were also enrolled and tested a mean (SD) of 2.13 (1.13) times each. CLWH were similar in age, sex, education, and number observations to HIV-negative controls (Table 1). All CLWH were receiving antiretroviral treatment.

### Comparison of Auditory Measures Between Groups

Using linear mixed-effects models, we evaluated the difference between HIV groups on static immittance, air-conduction thresholds, DPOAE amplitudes, and click-evoked ABR latencies and amplitudes. Mixed-effects models showed no difference between HIV groups on static immittance, or air-conduction thresholds as expected (eTable 1 and eFigure in Supplement 1). CLWH had significantly decreased DPOAE amplitudes at 6.0 and 8.0 kHz in left and right ears compared with HIV-negative controls. CLWH also had significantly reduced ABR wave V amplitude compared with the HIV-negative group for the slow and fast click rate. Table 2 and Table 3 show results for linear mixed-effects models for DPOAE amplitudes and ABR measures respectively. HIV status was independently associated with approximately 1.4 dB (95% CI, −3.28 to 0.30 dB) to 3.8 dB (95% CI, 6.03 to −1.99 dB) lower DPOAE amplitudes at 6 and 8 kHz bilaterally and 0.28 μV (95% CI, 0.01 to 0.33 μV) lower ABR wave V amplitudes in the right ear.

**Table 2.** Linear Mixed-Effects Models of DPOAEs for the Right and Left Ears

Ear	DPOAE signal frequency	Mixed-effects model	
		Estimate (95% CI)	*P* value
Right	1500 Hz	−0.451 (−1.941 to 1.038)	.55
	2000 Hz	0.232 (−1.836 to 1.371)	.77
	3000 Hz	−0.799 (−2.295 to 0.697)	.29
	4000 Hz	−0.301 (−1.846 to 1.244)	.70
	6000 Hz^a^	−1.492 (−3.285 to 0.301)	.00
	8000 Hz^a^	−3.814 (−6.034 to −1.994)	<.001
Left	1500 Hz	0.111 (−1.560 to 1.782)	.89
	2000 Hz	0.414 (−1.151 to 1.979)	.60
	3000 Hz	−0.535 (−1.896 to 0.826)	.44
	4000 Hz	−1.924 (−3.496 to −0.352)	.01
	6000 Hz^a^	−1.509 (−3.193 to 0.310)	.00
	8000 Hz^a^	−3.618 (−6.259 to −1.265)	<.001

Abbreviations: CLWH, children living with HIV; DPOAEs, distortion product otoacoustic emissions.

^a^ DOPAE amplitude values were significantly different between HIV groups at the *P* < .005 level for the mixed-effects models.

**Table 3.** Linear Mixed-Effects Models of ABR Amplitude and Latency Component Measures in the Right Ear

Click rate	Measure	Component	Mixed-effects model	*P* value
			Estimate (95% CI)	
Slow	Amplitude	I	−0.127 (−0.191 to 0.001)	.01
		III	−0.051 (−0.076 to 0.004)	.047
		V^a^	−0.290 (−0.327 to −0.019)	.003
	Latency	I	−0.009 (−0.110 to 0.093)	.86
		III	−0.058 (−0.150 to 0.035)	.22
		V	0.020 (−0.080 to 0.120)	.69
Fast	Amplitude	I	−0.006 (−0.024 to 0.012)	.49
		III	−0.042 (−0.098 to 0.001)	.04
		V^a^	−0.278 (−0.269 to −0.011)	.004
	Latency	I	0.021 (−0.099 to 0.141)	.73
		III	0.044 (−0.057 to 0.145)	.39
		V	0.036 (−0.071 to 0.143)	.51

Abbreviations: ABR, auditory brainstem responses; CLWH, children living with HIV.

^a^ ABR wave V amplitude using a slow click (21.1/s) and fast click (61.1/s) were significantly reduced in CLWH compared with HIV-negative controls.

**Figure 1.**
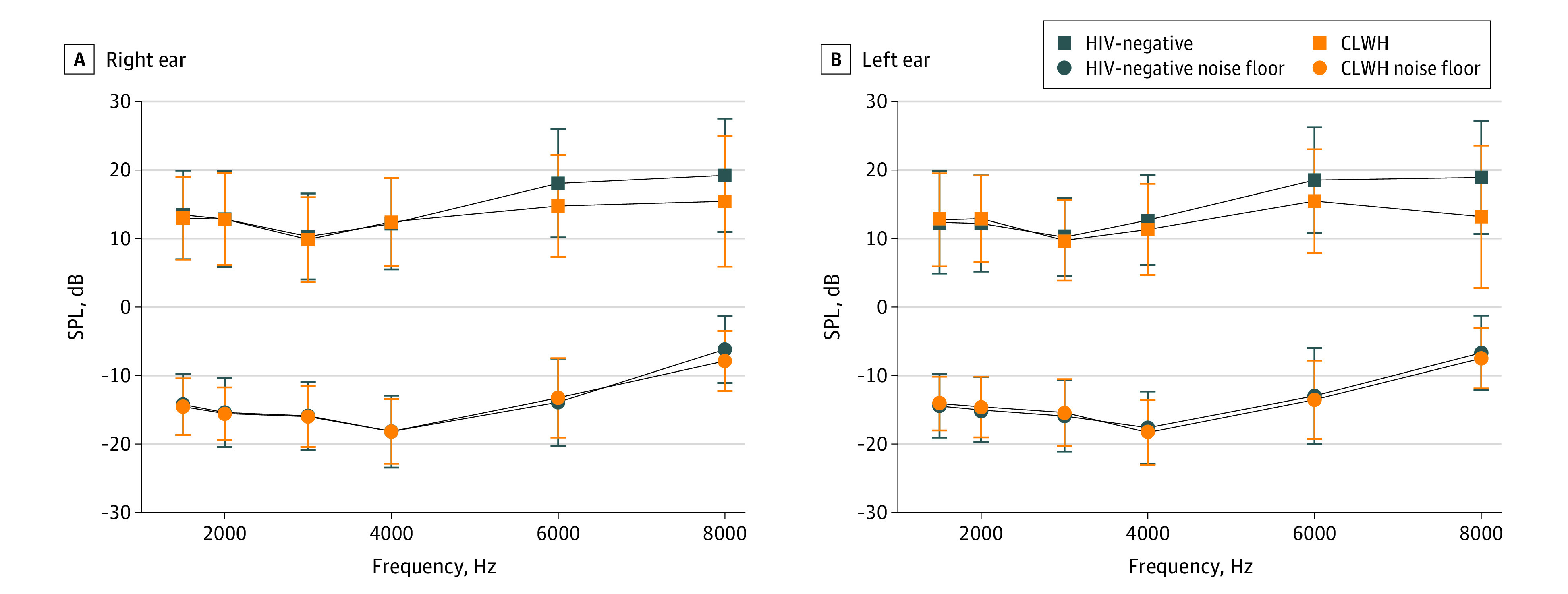
DPOAE Amplitudes and Noise Floors Plotted for Each Measured Frequency The top panel shows distortion product otoacoustic emissions (DPOAEs) for the right ear and the bottom panel shows DPOAEs for the left ear. Error bars indicate SD. CLWH indicates children living with HIV; SPL, sound pressure level.

**Figure 2.**
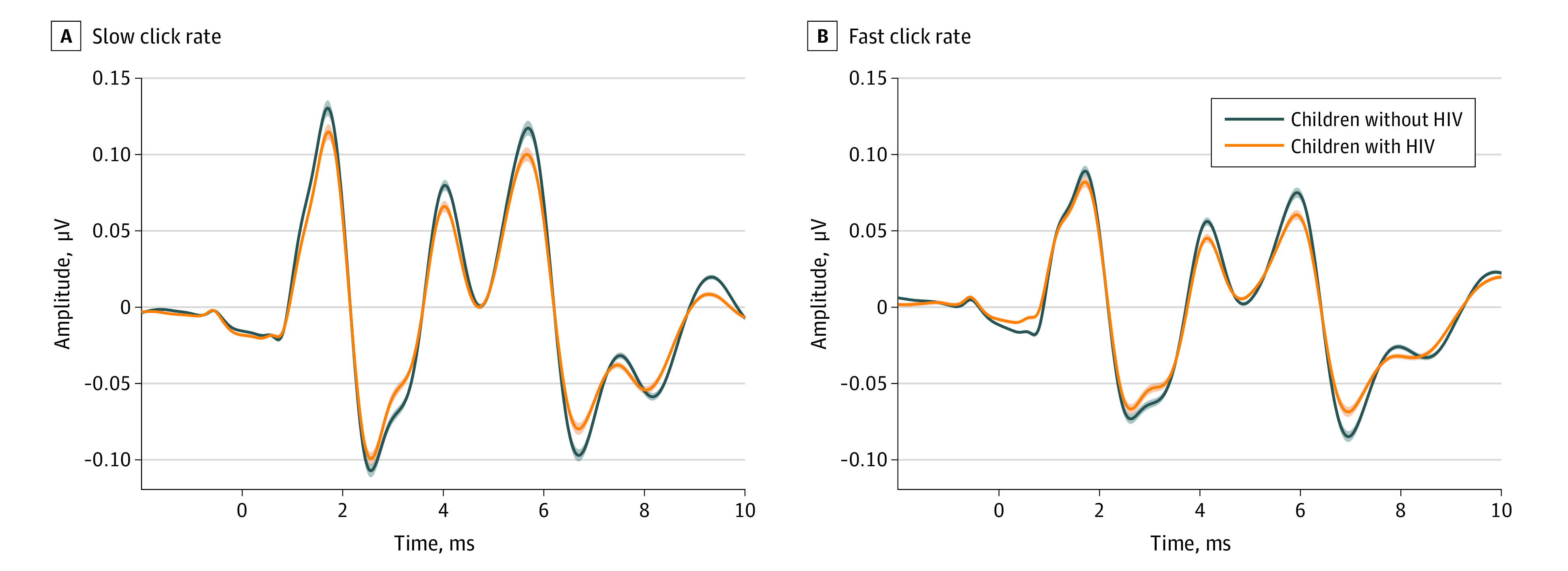
Auditory Brainstem Responses Grand Means for HIV Groups The left panel shows the slow click rate, and the right panel shows the fast click rate. HIV-negative participants are plotted in blue and CLWH are plotted in orange.

Figure 1 and Figure 2 illustrate differences between groups on DPOAE and ABR measures. Figure 1 shows lower DPOAE amplitudes in CLWH at 6 and 8 kHz in both ears. The large signal to noise ratios for both groups indicate high quality DPOAE recordings. Figure 2 shows ABR grand means for both groups in the right ear (ABRs were not conducted in the left ear). These panels show typical morphology of ABR component peaks with a significantly smaller wave V amplitude in CLWH compared with the HIV-negative group for both the slow and fast click rate. A nonsignificant reduction in amplitude across all peaks was observed for CLWH.

### Supplementary Antiretroviral Regimen Analysis

We conducted a brief supplementary analysis on common antiretroviral therapy regimens that were prescribed to CLWH. Our cohort is predominately on 2 antiretroviral regimens: abacavir, lamivudine, and lopinavir/ritonavir; and abacavir, lamivudine, and dolutegravir. Linear mixed-effects models of antiretroviral drug regimens in CLWH at initial visit and at final visit were conducted to assess the association between antiretroviral therapy and DPOAE and ABR variables. We did not find any significant differences between 2 common antiretroviral drug regimens in CLWH at the initial or most recent visit (see eAppendix, eTable 2, and eTable 3 in Supplement 1 for more details).

## Discussion

We compared peripheral auditory function between CLWH and HIV-negative controls with clinically normal hearing. We hypothesized subclinical decreases in peripheral auditory function in the CLWH due to HIV infection or treatment effects on auditory function according to our previous work in a young adult cohort.^12^ Results from the current study are consistent with subtle but significant reductions in high frequency DPOAEs and ABR wave V amplitude in CLWH compared with an age- and sex-matched HIV-negative group. HIV-positive status was independently associated with approximately 0.4 to 3.8 dB lower DPOAE amplitudes at 6 and 8 kHz in both ears and approximately 0.28μV lower ABR wave V amplitudes in the right ear. Although the differences between HIV groups were subclinical, decreased auditory function could affect a child’s ability to develop speech, language, and social skills, which would have lifelong consequences.

These results are consistent with our previous work in young adults, aged 20 to 30 years,^12^ middle age adults, aged 37 to 49 years,^4^ and HIV-positive children aged 1 to 18 years^15^ living in Dar es Salaam, Tanzania. All previous studies found slightly, but reliably, smaller DPOAEs in HIV-positive individuals compared with HIV-negative individuals.^4,12^ In HIV-positive children, we found DPOAE reductions in CLWH, but also higher rates of ear drainage, abnormal tympanometry (Type B or C), and tuberculous infection.^15^ In this study we controlled for normal middle ear function, ear drainage, and hearing thresholds, and found reduced DPOAE amplitudes, suggesting a HIV-associated outcome. In the young adult study,^12^ HIV-positive status was independently associated with approximately lower DPOAE amplitudes from 2 to 8 kHz (95% CI, 1.01-6.82 kHz) in both ears and lower ABR wave I amplitude in the right ear. The findings from the current study are consistent with those in young adults in 2 ways: (1) the directionality of change is similar, with HIV-positive groups demonstrating smaller DPOAEs and ABR amplitude profiles than their HIV-negative counterparts, and (2) the only significant differences on the ABR are related to the amplitude. That is, no ABR latencies were significantly different between groups in either analysis.

There are, however, differences between the 2 studies. HIV-positive young adults showed impaired DPOAEs across a broader frequency range than HIV-positive children. This could potentially be due to deterioration of the outer hair cells of the cochlea due to age. Also, the statistically significant components of the ABR profile also differed between the 2 studies. In the young adults, wave I was significantly smaller in people living with HIV; in the children, wave V amplitude was smaller. This could be due to multiple factors. First, the corrected α level (*P* = .005) to assess significance in this study was more conservative compared with the young adult study (*P* = .05) due to multiple comparisons. Likewise, visual analysis of Figure 2 shows a lower wave I amplitude in the CLWH compared with the HIV-negative group. Second, since wave V of the ABR generally reflects processing at the lateral lemniscus and inferior colliculus,^16,17,18^ we could interpret the wave V deficit as suggestive of HIV’s association with the development of distal structures in the central auditory pathway. The maturation of the ABR wave V amplitude increases monotonically through 6 months of age, then increases abruptly, reaching a plateau at 2 to 4 years.^19,20^ Given the mean age of both groups (approximately 7.2 years), it is unlikely that differences between groups are due to maturation but are consistent with subclinical dysfunction in central auditory pathways in HIV.^2,8,21^

Since HIV affects the central nervous system, it is possible that HIV directly affects the central auditory pathways.^21,22,23^ HIV does not invade neurons directly but can still cause indirect damage via inflammation and glial cell infection.^24^ HIV can also be detected in the central nervous system as early as 8 days after exposure, meaning any effects on the central nervous system could be immediate and evidenced in a pediatric population.^24^ In a review by Jong et al,^25^ HIV-positive individuals showed overall decreased DPOAEs and sensorineural hearing loss related to progression of disease and with older age. Central auditory measurements such as gap detection thresholds, ABR, and auditory electrophysiologic tests (ie, MLR and P300) also showed central auditory involvement among people living with HIV.^25^ They attributed these deficits to increased immune activation, inflammation, and degradation in the brain, caused by HIV. Even when prescribed an antiretroviral drug regimen, HIV-positive individuals may still experience neurological and cognitive symptoms.^25^ Since many antiretroviral therapies cannot readily cross the blood brain barrier, HIV may be allowed to persist in the brain and perhaps the cochlea.^26^ If uncontrolled, the virus could also plausibly damage important central auditory structures and pathways.^27^

Effects on DPOAEs could also be due to changes in the central nervous system. Our previous studies have shown that HIV affects the brain and central auditory processing.^2,11,21^ This could affect efferent auditory pathways, which directly influence outer hair cell motility and potentially DPOAE amplitudes.^28^ The efferent auditory pathway enhances the detection of speech perception in noise^29,30^ and maintains efficiency of cochlear function (ie, traveling wave).^31,32^ A reduction in efferent activity could help explain why people with HIV report hearing difficulties and show reduced DPOAEs, yet typically have normal audiometric thresholds.^4^ These subclinical hearing difficulties may be associated with learning difficulties for CLWH compared with their HIV-negative peers. This possibility is supported by other work documenting impaired cognition in CLWH.^33^ Future research on how the efferent auditory pathway affects DPOAEs in other diseases that affect the central nervous system is warranted.

The cochlea may also act as a reservoir for HIV potentially leading to subclinical peripheral deficits detected in our cohort. Mixed results have shown variable HIV effects on the cochlea with no consensus on a determining factor.^1,2,5,10,12,25,34^ Among these factors, accumulation of antiretroviral therapies could have also been associated with direct damage to the cochlea in the current study.^35,36^ Antiretroviral therapies and other potentially ototoxic drug therapies are hypothesized to damage the cochlea by destroying inner and outer hair cells, typically starting at the base and progressing toward the apex.^37^ We conducted a brief supplementary analysis on common antiretroviral therapy regimens that were prescribed to CLWH. We did not find any significant differences between 2 common antiretroviral drug regimens in CLWH at the initial or most recent visit (see eAppendix, eTable 2, and eTable 3 in Supplement 1 for more details). If antiretroviral therapy duration affected results, we would expect to see the subclinical deficits on DPOAEs across a broader frequency range (ie, affecting the whole cochlea) in young adults than in children, which is consistent with the current results. A previous study, however, showed lower DPOAE amplitudes in HIV-positive children, across a wider frequency range (differences were significant from 1.4–7.99 kHz) than in the present study.^15^ Although this previous study did not control for normal hearing sensitivity, it supports DPOAE magnitude differences between CLWH and HIV-negative children. In another previous study, we examined a cohort of middle-aged adults living with HIV who started antiretroviral therapy while they were in the study. This offered the unique opportunity to determine the outcomes of starting antiretroviral therapy. Starting antiretroviral therapy did lead to an increased rate of decline in DPOAE amplitudes, but this rate of decline was the same as in HIV-negative individuals, leading to an inconclusive result.^2^ These mixed findings suggest the need for additional testing of DPOAEs in children and adults with HIV to understand the outcomes of HIV and antiretroviral treatment across the lifespan.

These subclinical hearing difficulties may be associated with learning difficulties for CLWH compared with their HIV-negative peers. This possibility is supported by other work documenting impaired cognition in CLWH.^33^ Future research in the efferent auditory pathway concerning diseases that affect the central nervous system is warranted and will help detail the functional consequences of these observed deficits.

### Limitations

Generalizability of this study is limited. The children recruited for this study are all from Tanzania and within a narrow age range (3-9 years). All CLWH were receiving antiretroviral therapy, which limits our ability to attribute these subclinical deficits to HIV, as opposed to an outcome of antiretroviral therapy. However, with our supplementary analysis, we are reasonably confident that one specific antiretroviral therapy was not the cause of HIV-positive related differences on DPOAE or ABR results. The detected DPOAE and ABR deficits are also subclinical, and it is unclear how these translate to functional deficits. Yet, these findings have now been detected across an age range of 1 to 60 years, strengthening validity. Therefore, these subclinical differences in peripheral auditory function could be part of the causal story explaining the higher incidence of hearing complaints in HIV-positive populations.^2^

## Conclusions

In summary, we found subtle but reliable differences in peripheral auditory function in CLWH compared with age- and sex-matched HIV-negative controls. Specifically, we found similar pure-tone thresholds and tympanometry, but reduced DPOAE amplitudes and reduced ABR wave V in CLWH compared with HIV-negative controls. Although these associations were small, they were statistically significant and consistent with previous results in children, young adults, and middle-aged adults with HIV. Work is underway to characterize the functional consequences of these subclinical deficits. These subclinical auditory deficits in CLWH highlight the importance of characterizing any effects on cognitive and literacy development. Long-term studies are also needed to understand how these subtle deficits in peripheral auditory function evolve in combination with concurrent drug therapies, comorbid conditions, and aging.

^b^The socioeconomic status values result from a principal component analysis that accounts for various SES factors used in our previous work.^33^

## Notes

Supplement 1. **eFigure.** Hearing Thresholds **eTable 1. **Linear Mixed-Effects Models of Static Admittance and Pure-Tone Audiometry From 0.5-8.0 kHz for the Right and Left Ear **eAppendix. **Supplementary Antiretroviral Regimen Analysis for CLWH **eTable 2. **DPOAE Linear Mixed-Effects Models of Antiretroviral Drug Regimens in CLWH at Initial Visit and at Final Visit **eTable 3. **ABR Linear Mixed-Effects Models of Antiretroviral Drug Regimens in CLWH at Initial Visit and at Final Visit

Supplement 2.
Data Sharing Statement
