# Supplementary material for: Peripheral Auditory Function in Tanzanian Children Living With HIV With Clinically Normal Hearing
Source: JAMA Netw Open. 2023 Mar 15;6(3):e233061. doi: 10.1001/jamanetworkopen.2023.3061 (PMC10018326; doi:10.1001/jamanetworkopen.2023.3061)
Supplement: Supplement 1. — eFigure. Hearing Thresholds eTable 1. Linear Mixed-Effects Models of Static Admittance and Pure-Tone Audiometry From 0.5-8.0 kHz for the Right and Left Ear eAppendix. Supplementary Antiretroviral Regimen Analysis for CLWH eTable 2. DPOAE Linear Mixed-Effects Models of Antiretroviral Drug Regimens in CLWH at Initial Visit and at Final Visit eTable 3. ABR Linear Mixed-Effects Models of Antiretroviral Drug Regimens in CLWH at Initial Visit and at Final Visit [file jamanetwopen-e233061-s001.pdf]

Supplemental Online Content

Niemczak CE, Ealer C, Fellows A, et al. Peripheral auditory function in Tanzanian children living with HIV with clinically normal hearing. *JAMA Netw Open*. 2023;6(3):e233061. doi:10.1001/jamanetworkopen.2023.3061

eFigure. Hearing Thresholds
eTable 1. Linear Mixed-Effects Models of Static Admittance and Pure-Tone Audiometry From 0.5-8.0 kHz for the Right and Left Ear
eAppendix. Supplementary Antiretroviral Regimen Analysis for CLWH
eTable 2. DPOAE Linear Mixed-Effects Models of Antiretroviral Drug Regimens in CLWH at Initial Visit and at Final Visit
eTable 3. ABR Linear Mixed-Effects Models of Antiretroviral Drug Regimens in CLWH at Initial Visit and at Final Visit

This supplemental material has been provided by the authors to give readers additional information about their work.

© 2023 Niemczak CE et al. *JAMA Network Open.*

eFigure 1. Hearing Thresholds

Pure-tone thresholds (audiogram format) are displayed from 0.5-8 kHz with inlayed static admittance measures. HIV-negative are plotted in black and CLWH are plotted in red. Error bars show ±1 standard deviation. Observed pure-tone audiometry results are consistent with normal peripheral hearing ability with all mean threshold values less than 20dB HL. Yet, CLWH showed small non-significant reductions in thresholds at 8.0kHz in both ears.

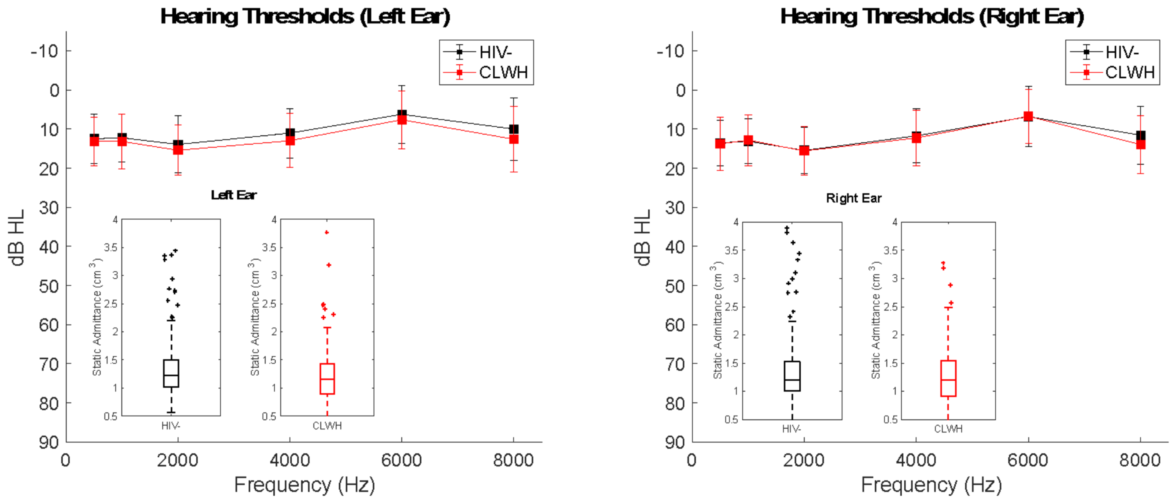

© 2023 Niemczak CE et al. *JAMA Network Open.*

eTable 1. Linear Mixed-Effects Models of Static Admittance and Pure-Tone Audiometry From 0.5-8.0 kHz for the Right and Left Ear

Model estimates reference the HIV-negative group (Model specification (*Measure ~ HIV status + (1|subject)*). No significant difference in static admittance or pure-tone audiometry existed between groups at a corrected *p*-value of 0.005 for the mixed-effects models.

Ear	Measure	Mixed-Effects Model	
		**Estimate (95% CI)**	***p*-Value**
**Right**	**Static Admittance**	-0.040 (-0.235, 0.154)	0.684
	**500 Hz**	0.192 (-1.180, 1.565)	0.783
	**1000 Hz**	-0.280 (-1.581, 1.021)	0.672
	**2000 Hz**	0.020 (-1.355, 1.395)	0.977
	**4000 Hz**	0.478 (-1.083, 2.040)	0.547
	**6000 Hz**	0.775 (-0.877, 2.428)	0.357
	**8000 Hz**	2.355 (0.699, 4.011)	0.008
**Left**	**Static Admittance**	-0.078 (-0.268, 0.111)	0.415
	**500 Hz**	0.554 (-0.829, 1.936)	0.431
	**1000 Hz**	0.924 (-0.472, 2.321)	0.194
	**2000 Hz**	1.367 (-0.197, 2.931)	0.086
	**4000 Hz**	1.744 (0.246, 3.243)	0.023
	**6000 Hz**	2.184 (0.544, 3.824)	0.010
	**8000 Hz**	2.389 (0.514, 4.264)	0.007

© 2023 Niemczak CE et al. *JAMA Network Open.*

eAppendix. Supplementary Antiretroviral Regimen Analysis for CLWH

Linear mixed-effects models of antiretroviral drug regimens in CLWH at initial visit and at final visit were conducted to assess the effect of antiretroviral therapy on DPOAE and ABR variables. Model estimates reference the antiretroviral regimen group at initial visit (Model #1 specification (*Measure* ~ ***Initial*** Antiretroviral *Drug Regimen* + *(1|subject)*) and at the final visit (Model #2 specification (*Measure* ~ ***Final*** Antiretroviral *Drug Regimen* + *(1|subject)*). The antiretroviral regimen at the subjects last (most recent) visit was predominantly one of two regimens: abacavir, lamivudine, lopinavir/ritonavir (38%), or abacavir, lamivudine, dolutegravir (31%). We coded antiretroviral regimens in one of three groups:

1. abacavir, lamivudine, lopinavir/ritonavir
2. abacavir, lamivudine, dolutegravir
3. other

But due to repeated visits, we also looked at the regimen CLWH were on at their initial visit, which indicated: abacavir, lamivudine, lopinavir/ritonavir (32%), or zidovudine, lamivudine, nevirapine (46%). We did a similar coding scheme for their initial visit:

1. abacavir, lamivudine, lopinavir/ritonavir
2. zidovudine, lamivudine, nevirapine
3. other

Since the average number of visits for CLWH was 2.23, we decided to run two sets of mixed-effects models, one on the initial antiretroviral regimen and the other on the current (or final visit) antiretroviral regimen. Results are shown below and in the supplementary analysis:

© 2023 Niemczak CE et al. *JAMA Network Open.*

eTable 2. DPOAE Linear Mixed-Effects Models of Antiretroviral Drug Regimens in CLWH at Initial Visit and at Final Visit

Ear	DPOAE Frequency	Initial Antiretroviral Regimen		Final Antiretroviral Regimen	
		**Estimate (95% CI)**	***p*-Value**	**Estimate (95% CI)**	***p*-Value**
**Right**	**1500 Hz**	0.232 (-1.257, 1.721)	0.758	-0.691 (-1.847, 0.465)	0.239
	**2000 Hz**	-0.024 (-1.680, 1.632)	0.977	-0.856 (-2.129, 0.417)	0.186
	**3000 Hz**	0.517 (-1.081, 2.116)	0.523	-0.751 (-1.910, 0.408)	0.202
	**4000 Hz**	-0.116 (-1.725, 1.493)	0.887	-1.000 (-2.197, 0.197)	0.101
	**6000 Hz**	0.093 (-1.722, -1.909)	0.919	-0.789 (-2.149, 0.570)	0.253
	**8000 Hz**	0.423 (-2.024, 2.869)	0.733	-0.612 (-2.455, 1.230)	0.512
**Left**	**1500 Hz**	-0.290 (-2.019, 1.439)	0.741	-1.546 (-2.777, -0.315)	0.014
	**2000 Hz**	-0.387 (-1.960, 1.187)	0.628	-1.606 (-2.794, -0.418)	0.010
	**3000 Hz**	-0.687 (-2.141, 0.767)	0.352	-0.912 (-2.024, 0.201)	0.107
	**4000 Hz**	-0.627 (-2.345, 1.091)	0.472	-1.081 (-2.303, 0.141)	0.082
	**6000 Hz**	0.977 (-0.968, 2.922)	0.322	-1.418 (-2.938, 0.102)	0.067
	**8000 Hz**	0.777 (-1.831, 3.384)	0.557	-1.105 (-3.077, 0.868)	0.270

© 2023 Niemczak CE et al. *JAMA Network Open.*

eTable 3. ABR Linear Mixed-Effects Models of Antiretroviral Drug Regimens in CLWH at Initial Visit and at Final Visit

Click Rate	Measure	Component	Initial Antiretroviral Regimen		Final Antiretroviral Regimen	
			Estimate (95% CI)	*p*-Value	Estimate (95% CI)	*p*-Value
Slow	Amplitude	I	-0.013 (-0.041, 0.015)	0.348	-0.002 (-0.025, 0.021)	0.850
		III	0.007 (-0.014, 0.028)	0.500	0.001 (-0.016, 0.017)	0.951
		V	0.012 (-0.012, 0.035)	0.332	-0.004 (-0.023, 0.015)	0.673
	Latency	I	-0.053 (-0.171, 0.064)	0.373	-0.004 (-0.098, 0.091)	0.936
		III	-0.075 (-0.176, 0.027)	0.148	0.011 (-0.073, 0.094)	0.796
		V	-0.030 (-0.141, 0.080)	0.588	0.033 (-0.058, 0.124)	0.472
Fast	Amplitude	I	-0.014 (-0.035, 0.007)	0.195	0.001 (-0.016, 0.018)	0.891
		III	-0.003 (-0.021, 0.015)	0.740	-0.007 (-0.021, 0.008)	0.353
		V	0.010 (-0.006, 0.025)	0.216	-0.002 (-0.015, 0.010)	0.698
	Latency	I	-0.076 (-0.216, 0.064)	0.283	-0.019 (-0.133, 0.095)	0.738
		III	-0.022 (-0.142, 0.098)	0.713	0.044 (-0.050, 0.138)	0.359
		V	-0.045 (-0.161, 0.070)	0.438	0.075 (-0.017, 0.167)	0.107

These analyses did not show a strong relationship between antiretroviral therapy and the difference in auditory variables between CLWH and HIV-negative children.

© 2023 Niemczak CE et al. *JAMA Network Open.*
