# Supplementary material for: Peripheral Auditory Function in Tanzanian Children Living With HIV With Clinically Normal Hearing
Source: JAMA Netw Open. 2023 Mar 15;6(3):e233061. doi: 10.1001/jamanetworkopen.2023.3061 (PMC10018326; doi:10.1001/jamanetworkopen.2023.3061)
Supplement: Supplement 2. — Data Sharing Statement [file jamanetwopen-e233061-s002.pdf]

Data Sharing Statement

Niemczak. Peripheral Auditory Function in Tanzanian Children Living With HIV With Clinically Normal Hearing. *JAMA Netw Open*. Published March 15, 2023.
doi:10.1001/jamanetworkopen.2023.3061

Data
Data available: No

Additional Information
Explanation for why data not available: The data will be made available upon request.
